# Chronic and acute mediators of passive viscoelasticity in human skeletal muscle fibres

**DOI:** 10.1113/EP092361

**Published:** 2025-05-26

**Authors:** Grace E. Privett, Austin W. Ricci, Karen Wiedenfeld Needham, Damien M. Callahan

**Affiliations:** ^1^ Department of Human Physiology University of Oregon Eugene Oregon USA

**Keywords:** cellular stiffness, fatigue, passive mechanics, skeletal muscle, stress decay, titin, training, viscoelasticity

## Abstract

The cellular viscoelastic modulus in skeletal muscle tissue responds dynamically to chronic stressors, such as age and exercise. Passive tissue mechanics can also be sensitive to acute stimuli, such as mechanical loading and/or activation‐induced muscle fatigue. These insights are largely derived from preclinical studies of age and acute muscle activation. Therefore, we sought to understand the relative responsiveness of muscle cellular passive mechanics to chronic (resistance training) and acute (exercise‐induced muscle fatigue) stressors in healthy young males and females categorized as ‘resistance trained’ or ‘untrained’. We measured passive mechanics to test the hypothesis that Young's modulus and stress would be greater in fibres from trained versus untrained participants and that both would be reduced following fatigue. We also assessed the translation of these findings to composite tissue in a subset of volunteers where muscle tissue bundles, containing both fibres and extracellular matrix, were analysed in addition to single fibres. We found that resistance‐trained individuals demonstrated enhanced passive elastic and viscous modulus compared with non‐trained individuals. We also report reductions in passive mechanical measures following fatiguing exercise. Surprisingly, both chronic and acute effectors of passive mechanics were observed in muscle fibres only from males, whereas females showed a more variable response across conditions. Last, we provide preliminary evidence supporting the translation of per‐individual cellular differences to the tissue level. Together, these data suggest that males respond more dynamically to acute and chronic stressors of muscle tissue mechanics, potentially linking cellular response and sex‐dependent differences in functional outcomes across the lifespan.

## Abstract (highlights)

- **What is the central question of this study?**
  Do acute mediators of human skeletal muscle viscoelasticity (fatigue) scale to those previously demonstrated by chronic effectors (sex and resistance training) of viscoelasticity, and are they affected by biological sex?
- **What is the main finding and its importance?**
  Fatiguing exercise altered passive stress and modulus to a greater extent than chronic effectors of passive skeletal muscle mechanics. However, fatigue effects were driven entirely by males, whereas responses in females were variable, with no net change. We find that fibre mechanics might contribute to multicellular muscle tissue, suggesting relevance to in vivo conditions.

## 1 INTRODUCTION

Skeletal muscle viscoelasticity affects functional mobility through multiple interrelated mechanisms, including its influence on joint stability (Blackburn et al., 2011) and the dampening of impact forces (Sarvazyan et al., 2014) during musculoskeletal loading. Viscoelastic behaviour of muscle also contributes to the rate and mechanical efficiency of force transfer from muscle to tendon (Wilson & Flanagan, 2008). Prior studies report that whole‐muscle stiffness and viscosity are transiently altered by a single bout of fatiguing exercise in the vastus lateralis (VL) (Chalchat et al., 2020). This transient change to VL mechanical properties might be maladaptive, considering that muscular fatigue was previously shown to decrease the capacity for skeletal muscle to absorb strain elastic energy before incurring injury (Mair et al., 1996). As such, acute, fatigue‐induced changes to skeletal muscle viscoelasticity might contribute to the high prevalence of hamstring injury in male athletes (Cross et al., 2013; Watsford et al., 2010) or knee‐joint destabilization (Blackburn et al., 2011) and subsequent anterior cruciate ligament injury in female athletes (De Ste Croix et al., 2015; Myer et al., 2008). Given the likely relationship between muscle viscoelastic properties and tissue injury risk, it is logically appealing to predict that biological sex, muscle fatigue and injury risk share a complex dynamic contributing to sex disparity in rates of soft tissue injury.

At the cellular level, we have demonstrated fatigue‐induced reductions in passive Young's modulus, a measure of stiffness accounting for differences in cell (‘fibre’) size, in VL samples from young, recreationally active adults. Notably, these acute, fatigue‐induced reductions were dependent on sex, in that the response was observed only in males (Privett et al., 2024). At the single‐fibre level, passive modulus is largely attributed to the intracellular protein titin (Lim et al., 2019; Ottenheijm et al., 2012). Titin is a viscoelastic protein that is subject to post‐translational modifications (PTMs), many of which can alter titin‐based stiffness if they occur at an extensible region of the protein (Hamdani et al., 2017). Ample study of PTM‐induced changes to titin‐based mechanical properties has been conducted in cardiac muscle, which has demonstrated that phosphorylation of cardiac titin regulates cardiomyocyte mechanics in health and disease (Fukuda et al., 2005; Granzier & Irving, 1995; Krüger et al., 2009; LeWinter & Granzier, 2014; Wu et al., 2002, 2000; Yamasaki et al., 2002). Skeletal muscle titin can also be phosphorylated (Müller et al., 2014; Privett et al., 2024), although whether titin phosphorylation alters viscoelasticity in skeletal muscle is not yet clear. Exercise is a multimodal and dynamic physiological stimulus that acutely induces multiple PTMs across multiple residues in a range of proteins that comprise skeletal muscle (Hamdani et al., 2017). These include phosphorylation (Müller et al., 2014), *S*‐glutathionylation (Alegre‐Cebollada et al., 2014; Watanabe et al., 2020) and the binding of small heat shock proteins (HSPs) (Kötter et al., 2014), the last of which has been shown to protect against aggregation of titin immunoglobulin domains and subsequent stiffening of the protein (Kötter et al., 2014). Evidence suggests that titin directly influences whole‐muscle function (Brynnel et al., 2018), supporting the clinically relevant translation of our recent observation that acute regulation of titin might influence skeletal muscle mechanics (Privett et al., 2024). Therefore, in the present study we sought to test the role of intracellular contributors to changes in tissue‐level mechanical properties by comparing the impact of acute fatigue on viscoelastic properties of single fibres (titin‐based) and in bundles of fibres with intact extracellular matrix (ECM) whose modulus is presumably impacted by several factors outside the myofibre.

Although the study of altered VL mechanical properties following fatiguing exercise might have high clinical relevance for athletes, it remains to be seen whether the neuromuscular adaptations to chronic training mediate these observations. Chronic training has been demonstrated to increase active (Mongold et al., 2022) and resting (Klinge et al., 1997) musculoskeletal stiffness in some, but not all (Blazevich, 2019), studies. Evidence of training‐based changes to collagen expression, synthesis, and accumulation in the ECM (Kjær, 2004) suggests that altered skeletal muscle stiffness attributable to training might arise from modified ECM‐based stiffness. Although it is unclear whether chronic training impacts ECM‐based measures of stiffness in the literature, jump training in rats has been shown to increase the passive stiffness of extensor digitorum longus and rectus femoris muscles in conjunction with increased collagen concentration (Ducomps et al., 2003). At the cellular level, training was shown to alter the stiffness of permeabilized skeletal muscle fibres in a length‐dependent manner (Noonan et al., 2020), suggesting that intracellular mechanisms might also contribute to training‐based changes in skeletal muscle stiffness. Therefore, in the present study we compared baseline measures of cellular passive viscoelasticity in VL samples from untrained (UT) and resistance‐trained (RT) young males and females and expanded upon our previous work (Privett et al., 2024) by assessing whether chronic training mediates the effect of fatiguing exercise on cellular passive mechanics.

Although the majority of previous research focused on measures of elasticity, viscosity is equally important to consider given its role in absorption and dissipation of skeletal muscle mechanical loads (Sarvazyan et al., 2014) during dynamic loading associated with repeated stretch–shortening cycles. Therefore, in the present study we sought to extend our previous work by considering the effect of fatigue on the viscoelasticity of human skeletal muscle cells. To do so, the stress decay index (SDI) was calculated using the magnitude and rate of stress relaxation at each sarcomere length (SL) studied.

The purpose of this study was to compare passive viscoelastic properties in VL samples obtained immediately after fatiguing, single‐joint, dynamic exercise (‘fatigued’) and non‐exercised, control (‘non‐fatigued’) limbs of UT and RT young adults. We hypothesized that passive mechanical measures (stress, Young's modulus) would be reduced in fatigued versus non‐fatigued skeletal muscle fibres from all participants. Prior studies suggest that resistance training increases the passive modulus (Noonan et al., 2020). Therefore, we also hypothesized that cellular passive mechanical measures would be higher in RT versus UT individuals. To consider the ‘viscous’ element of skeletal muscle viscoelasticity, SDI was compared in fatigued and non‐fatigued skeletal muscle cells. Finally, the passive modulus was measured in bundles of fibres with intact ECM, here termed ‘composite muscle tissue’ (CMT), before and after chemical elimination of intracellular contributors to passive modulus to assess the extent to which intracellular mechanisms contribute to observations of altered CMT passive modulus after fatiguing exercise. It was hypothesized that any effect of fatiguing exercise on measures of CMT stiffness would be abolished by elimination of intracellular contributors to passive modulus.

## 2 MATERIALS AND METHODS

### 2.1 Ethical approval

The study protocol was approved by the Institutional Review Board at the University of Oregon and conformed to the standards set by the *Declaration of Helsinki*. Although completed prior to the 2024 revision to the *Declaration of Helsinki*, all aspects of the protocol were consistent with changes in the latest revision. Prior to participation, all volunteers provided written informed consent.

### 2.2 Population

Nineteen young (aged 21 ± 3 years), RT and UT males and females from the University of Oregon and surrounding community consented to participate in this study. Participants in the RT group reported between five and seven weekly sessions of resistance training. Each was ≥1 h in duration, and at least three sessions focused on the lower extremities. Participants in the UT cohort reported a complete lack of structured physical exercise and no resistance training. Self‐reported physical activity was confirmed using ActivePal accelerometers (Glasgow, UK), as described previously (Dowd et al., 2012). To limit the potential for menstrual cycle‐dependent variation in circulating estradiol and potential impacts on skeletal muscle mechanical properties, all eumenorrhoeic female volunteers were tested in the prefollicular phase of the menstrual cycle, (within 5 days of menses onset). Participants using hormonal contraceptive were expected to experience consistent oestrogen suppression. Therefore, participation dates were scheduled without regard for cyclical variations in free estradiol. Participants reported no orthopaedic limitations (severe osteoarthritis, joint replacement or other orthopaedic surgery in the previous 6 months), endocrine disease (hypo/hyperthyroidism, Addison's disease or Cushing's syndrome), uncontrolled hypertension (>140/90 mmHg), neuromuscular disorder, significant heart, liver, kidney or respiratory disease or diabetes. Participants were non‐tobacco smokers and had no current alcohol disorder. Finally, participants taking medications known to affect muscle stiffness or β‐adrenergic signalling of neuromuscular activation (including but not limited to β‐blockers, calcium channel blockers and muscle relaxants) or anabolic steroids were not included.

### 2.3 Study design

Participants visited the laboratory on two occasions separated by ≥1 week. Both visits were scheduled between 08.00 and 10.00 h to control for diurnal variations that might impact skeletal muscle and the response to exercise. Participants were instructed to arrive in the fasted state and having consumed no caffeine. Water consumption was encouraged. During the first visit, participants were familiarized with the exercise protocol while non‐invasive measures of voluntary strength, power and fatigue of their dominant knee extensors were collected using a Biodex System 3 dynamometer (Biodex Medical Systems, Shirley, NY, USA). During the second visit, volunteers performed maximal voluntary isometric contractions of the knee extensors followed by fatiguing exercise to task failure. Fatiguing exercise was followed by bilateral, percutaneous needle muscle biopsies: one on the exercised limb immediately following exercise (‘fatigued’) and the second on the contralateral, non‐exercised limb (‘non‐fatigued’).

### 2.4 Fatigue protocol

Muscle fatigue was achieved as described in detail previously (Privett et al., 2024). Briefly, participants were seated in the dynamometer, with hips and knees flexed at 90° (180° = full extension). After completion of three maximum voluntary isometric contractions of the dominant knee extensors, participants performed repeated, voluntary knee extensions against isotonic resistance equivalent to 30% of maximum voluntary isometric contraction until task failure. Task failure was identified as the inability to perform knee extension through ≥50% of the range of motion. Fatigue was quantified as the fatigue ratio (final power divided by initial power, where ‘initial power’ represents the average peak power of the first five knee extensions performed during fatiguing exercise, and ‘final power’ represents the average peak power from the last five knee extensions). Time to fatigue (task failure) was recorded for all participants.

### 2.5 Muscle biopsy procedure

Percutaneous needle biopsy of the VL was performed within 9 ± 4 min following task failure, in sterile conditions, as detailed previously (Tarnopolsky et al., 2011). Initially, the biopsy site was sterilized, and local anaesthetic (1% or 2% lignocaine hydrochloride; Hospira Worldwide, Lake Forest, IL, USA) was administered via injection. Next, a small (∼5 mm) incision was made in the skin and muscle fascia, allowing for the passage of a Bergström biopsy needle (5 mm in diameter) into the belly of the VL muscle to acquire sample at a depth of ∼2–3 cm. After acquisition of the biopsy sample, the collected muscle was retrieved from the needle using forceps.

### 2.6 Tissue processing and dissection

The details of tissue processing for mechanics were detailed elsewhere (Privett et al., 2024). Briefly, the sample collected during biopsy was placed in muscle dissecting solution [MDS; 120.782 mM sodium methanesulphonate (NaMS), 5.00 mM EGTA, 0.118 mM CaCl_2_, 1.00 mM MgCl_2_, 5.00 mM ATP‐Na_2_H_2_, 0.25 mM KH_2_PO_4_, 20.00 mM BES, 1.789 mM KOH and 1 mM dithiothreitol (DTT)], parsed into bundles of ∼50 fibres, and tied to glass rods before advancement through solutions of increasing glycerol content and storage in 50% glycerol solution (5.00 mM EGTA, 2.50 mM MgCl_2_, 2.50 mM ATP‐Na_2_H_2_, 10 mM imidazole, 170.00 mM potassium propionate, 1.00 mM sodium azide and 50% glycerol by volume) at −20°C. Samples allocated for mechanics analyses were used within 4 weeks following biopsy.

Preparation for mechanical assays was described previously (Privett et al., 2024). Briefly, fibre bundles and dissected single fibres were chemically skinned (MDS + 1% Triton X‐100), transferred to plain MDS and kept on ice until experimentation. For CMT experiments, fibre bundles were transferred directly from 50% glycerol solution to plain MDS (no further chemical skinning). Then, strips of 12–14 fibres with surrounding ECM were dissected from the bundle and kept on ice until experimentation.

### 2.7 Single‐fibre morphology and contractile measures

Prepared fibres were mounted in relaxing solution [67.286 mM NaMS, 5.00 mM EGTA, 0.118 mM CaCl_2_, 6.867 mM MgCl_2_, 0.25 mM KH_2_PO_4_, 20.00 mM BES, 0.262 mM KOH, 1.00 mM DTT, 5.392 mM Mg‐ATP, 15.00 mM creatine phosphate (CP) and 300 U/mL creatine phosphokinase (CPK)] between a force transducer and a length motor (Aurora Scientific, Inc., Aurora, ON, Canada) using the Moss clamp technique (Moss, 1979). Passive tension was measured by zeroing the force transducer while the fibre was slack, then stretching the fibre to SL = 2.65 µm. Active tension was measured at SL 2.65 µm by moving the fibre to pre‐activating solution (81.181 mM NaMS, 5.00 mM EGTA, 0.012 mM CaCl_2_, 6.724 mM MgCl_2_, 5.00 mM KH_2_PO_4_, 20.00 mM BES, 1.00 mM DTT, 5.397 mM Mg‐ATP, 15.00 mM CP and 300 U/mL CPK), followed by activating solution (57.549 mM NaMS, 5.00 mM EGTA, 5.021 mM CaCl_2_, 6.711 mM MgCl_2_, 5.00 mM KH_2_PO_4_, 20.00 mM BES, 9.674 mM KOH, 1.00 mM DTT, 5.437 mM Mg‐ATP, 15.00 mM CP and 300 U/mL CPK). Once a steady‐state tension was recorded, the sample was returned to relaxing solution. All fibres were activated (pCa 4.5) prior to passive stretching to measure active tension and confirm fibre viability.

### 2.8 Passive stretch protocol

**FIGURE 1:**
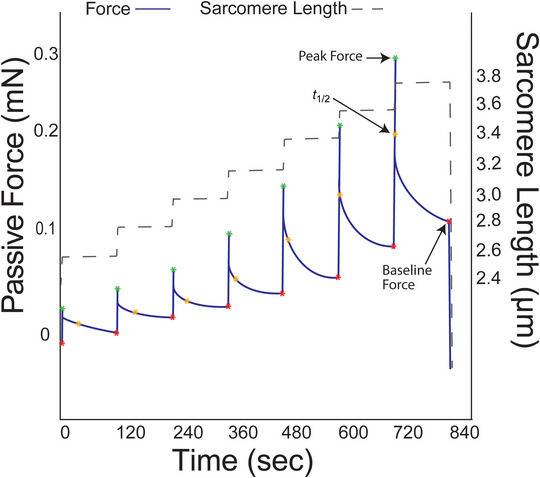
Sample force trace, indicating the outcome measures produced by this passive stretch protocol. Baseline force was used to calculate passive stress and peak force. Half‐relaxation time (*t*_½_) and baseline force were used to calculate the stress decay index.

Passive stretch measures were performed in relaxing solution (pCa 8.0) using a passive stretch protocol (Figure 1) adapted from previous work (Lim et al., 2019). Initial sarcomere length was set to 2.4 µm, followed by seven incremental stretches to reach a final length of 156% of initial length (SL = 3.5–4.0 µm). Each stretch lengthened the sample by 8% of initial length and held this position for 2 min of stress relaxation. The SL was measured throughout the protocol using an inverted microscope located beneath the single‐fibre rig. To determine the extent to which actomyosin interactions contributed to measures of passive viscoelasticity, a subset of fibres from two sedentary males was subjected to the passive stretch protocol in relaxing solution with the addition of 40 mM 2,3‐butanedione monoxime (BDM), a myosin inhibitor. Following completion of the passive stretch protocol, each fibre was collected and placed in gel loading buffer (2% SDS, 62.5 mM Tris, 10% glycerol, 0.001% Bromophenol Blue and 5% β‐mercaptoethanol, pH 6.8), centrifuged and heated at 65°C for 2 min, then stored at −80°C until later assessment of the myosin heavy chain (MHC) isoform.

### 2.9 Potassium iodide/potassium chloride treatment

To assess the contribution of titin to the passive modulus in CMT, mounted bundles of ∼12–14 fibres with intact ECM were treated with potassium chloride (KCl) and potassium iodide (KI) to extract the thick and thin sarcomeric filaments, respectively (Brynnel et al., 2018; Ottenheijm et al., 2012). CMT samples were first subjected to an initial passive stretch (as described above), after which the sample was incubated in relaxing solution containing 0.6 M KCl for 35 min at 15°C followed by relaxing solution containing 1.0 M KI for 35 min at 15°C. After incubation, CMT samples were passively stretched a second time to collect post‐treatment passive mechanical measures. After these experiments, CMT samples were collected and stored in gel loading buffer.

### 2.10 MHC isoform identification

**FIGURE 2:**
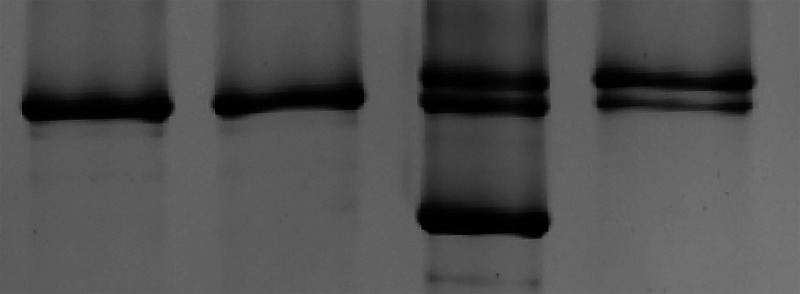
Sample image of silver‐stained MHC bands. The left lane shows a fibre exhibiting the MHC I isoform, the two middle bands exhibit the MHC IIA isoform, and right lane contains a sample homogenate used to visualize all three isoforms (I, IIA and IIX). Abbreviation: MHC, myosin heavy chain.

SDS–PAGE was used to determine the MHC isoform of single muscle fibres. A sample from each fibre was loaded into its own well of a 4% stacking–7% resolving polyacrylamide gel. The gel was run at 70 V for 3.5 h, followed by 200 V for 20 h at 4°C (Miller et al., 2010). Gels were stained with silver, and the resulting MHC isoform (I, IIA and/or IIX) expression was determined by comparison to a standard made from a multifibre homogenate (Figure 2).

### 2.11 Outcome measures

Maximally activated tension was quantified as the plateau of active force divided by fibre cross‐sectional area. Passive stress at each SL was calculated as baseline *F*/CSA, where ‘baseline *F*’ indicates force following stress relaxation (Figure 1), and ‘CSA’ indicates fibre cross‐sectional area assuming an elliptical shape. $\mathrm{CSA}=\pi (\frac{d_{\mathrm{top}}}{2}\times \frac{d_{\mathrm{side}}}{2})$, where ‘$d_{\mathrm{top}}$’ is the average of three top diameter measures, and ‘$d_{\mathrm{side}}$’ is the average of three side diameter measures. Strain was calculated as $\frac{\Delta L}{L_{0}}$, where ‘*L*_0_’ indicates initial length. Using raw stress data, the passive Young's modulus was calculated as the slope of the stress–strain relationship at shorter fibre lengths (strain = 1.0%–1.24 %*L*_0_) and at longer fibre lengths (strain = 1.32%–1.56%*L*_0_). For fibre measures of the passive modulus, separate slopes were calculated for shorter and longer fibre lengths to consider the length dependence of passive modulus measures. The stress decay index (SDI) was calculated as previously described (Lim et al., 2019): $\left(\mathrm{peak}\mathrm{stress}-\mathrm{stress}\right)\times \frac{t_{1/2}}{\log2}$, where peak stress is peak force/CSA and *t*_½_ is the half‐relaxation time (Figure 1). For tissue‐level measures, the passive Young's modulus was calculated as one slope of the stress–strain curve, given the lack of a clear curvilinear inflection point such as that observed in the stress–strain data of the fibres. The direction and magnitude of change in modulus between non‐fatigued and fatigued samples was calculated for fibre and CMT samples as F−NFμ/F−NFμNFμNFμ, where ‘*F*’ indicates the fatigued value for each individual sample, and ‘NF_μ_’ indicates the group mean of the non‐fatigued samples. For KI/KCl passive measures of CMT, the passive modulus was calculated as one slope of the stress–strain curve.

### 2.12 Statistical analyses

Statistical testing was conducted using the SPSS software package (SPSS, IBM Corp., Armonk, NY, USA) unless otherwise specified. At each SL, differences in passive stress and SDI were evaluated using separate linear mixed models including fatigue, biological sex, training and interaction terms as fixed effects, with participant identity as a random effect to account for fibre variation within individuals, as described previously (Callahan et al., 2015). To evaluate differences in single‐fibre passive modulus at short and long lengths, separate linear mixed models were run, with fatigue, biological sex, training and interaction terms as fixed effects and participant identity as a random effect. To test for differences in maximally activated tension, a mixed‐effects model was run, including sex, training and fatigue as main effects and participant identity as a random effect. Of note, because MHC IIA fibres were represented similarly across all groups, all statistical analyses for single‐fibre mechanics included only MHC IIA fibres. This decision precluded the consideration of interactions between fibre type and other main effects (sex/fatigue) in our cohort. Fibre number is limited compared with the total dataset (206 of 423 fibres in total), but we felt this to be the most rigorous and conservative approach to test our hypotheses with respect to sex and fatigue. To test for significant differences in the passive Young's modulus of CMT samples, a linear mixed model was generated, with biological sex and fatigue as fixed effects and participant as a random effect. Follow‐up analyses tested for an effect of fatigue, with participants as a random effect, on the passive modulus of CMT samples from males and females, separately. To test for fatigue‐based differences in CMT passive modulus before and after KI/KCl treatment, means for the four fatigue/treatment groups (non‐fatigued pre‐KI/KCl, non‐fatigued post‐ KI/KCl, fatigued pre‐ KI/KCl and fatigued post‐ KI/KCl) were compared in a univariate ANOVA, with least significance difference *post hoc* testing.

## 3 RESULTS

### 3.1 Descriptive measures

Males were taller than females (184.3 ± 8.6 vs. 161.6 ± 4.9 cm, respectively, *p <* 0.01) and weighed more than females (81.3 ± 9.3 vs. 57.4 ± 5.2 kg, respectively, *p <* 0.01; Table 1). There was no significant main effect of training on height (*p =* 0.691) or weight (*p =* 0.997). Body mass index was significantly higher in males versus females (23.9 ± 1.7 vs. 22.0 ± 1.8 kg m^−2^, respectively, *p =* 0.023), but was not different between RT and UT participants (*p =* 0.463). Participants in the RT cohort had greater physical activity, as expected (12 505 ± 2823 vs. 7201 ± 2162 steps, respectively, *p <* 0.01) and spent almost twice as much time per day in moderate physical activity (101.8 ± 19.3 vs. 54.0 ± 16.0 min per day, respectively, *p <* 0.01). There was no effect of biological sex on step count (*p =* 0.928) or time spent in moderate physical activity (*p =* 0.614). There was no significant main effect of training status or biological sex on the number of minutes spent in light physical activity (*p =* 0.090 and 0.185, respectively) or in vigorous physical activity (*p =* 0.183 and *p =* 0.400, respectively).

**TABLE 1:** Anthropometric and activity data of included participants.

Group	Sex	*n*	Height^*^ (cm)	Weight^*^ (kg)	Body mass index^*^ (kg m^−2^)	Step count^†^ (steps day^−1^)	Light activity (min day^−1^)	Moderate activity^†^ (min day^−1^)	Vigorous activity^†^ (min day^−1^)
Untrained	Female	5	162.1 ± 6.1	55.0 ± 3.2	20.9 ± 0.6	7274 ± 2162	27 ± 9	56 ± 18	0.5 ± 0.6
	Male	4	185.3 ± 13.0	84.0 ± 10.1	24.4 ± 1.2	7108 ± 1525	38 ± 7	51 ± 15	1.1 ± 1.2
Trained	Female	5	160.1 ± 3.9	60.0 ± 6.0	23.1 ± 2.1	12 357 ± 2531	40 ± 13	96 ± 16	5.0 ± 6.2
	Male	5	183.6 ± 4.5	79.1 ± 9.2	23.4 ± 2.1	12 752 ± 3864	43 ± 12	111 ± 24	1.5 ± 1.3

*Note*: Data are shown as the mean ± SD. Symbols indicate a significant main effect of biological sex (^*^)or training (^†^) between groups (*p <* 0.05).

### 3.2 Fatiguing exercise and whole‐muscle contractile measures

Owing to technical limitations related to data loss during transfer, time to fatigue and knee extensor power measures are reported for 18 of 19 and 16 of 19 volunteers, respectively. The average time to fatigue was not significantly different by training status (*p =* 0.453) or biological sex (*p =* 0.559). Likewise, there was no effect of training status (*p =* 0.415) or biological sex (0.926) on fatigue ratio (Table 3). Absolute peak power was significantly higher in RT versus UT (608.35 ± 295.87 vs. 413.84 ± 143.11 W, respectively, *p =* 0.017) and male versus female (683.13 ± 238.86 vs. 339.06 ± 70.27 W, respectively, *p <* 0.001) participants. Relative peak power was significantly higher in RT versus UT (8.27 ± 2.58 vs. 5.93 ± 1.09 W kg^−1^, respectively, *p =* 0.002) and male versus female (8.40 ± 2.60 vs. 5.81 ± 0.64 W, respectively, *p =* 0.001) participants. Absolute peak torque was significantly higher in RT versus UT (279.55 ± 102.78 vs. 202.45 ± 79.51 N, respectively, *p =* 0.004) and male versus female (327.32 ± 60.12 vs. 167.16 ± 50.97 N, respectively, *p <* 0.001) participants. Finally, relative peak torque was significantly higher in RT versus UT (3.91 ± 0.96 vs. 2.90 ± 0.48 N kg^−1^, respectively, *p =* 0.001) and male versus female (4.06 ± 0.80 vs. 2.87 ± 0.60 N kg^−1^, respectively, *p <* 0.001) participants.

### 3.3 Data integrity and quality control

In total, data were collected and analysed for 424 fibres (Table 2). Single‐fibre CSA was greater in males versus females (0.0074 ± 0.0024 vs. 0.0050 ± 0.0016 mm^2^, respectively, *p <* 0.01) and greater in RT versus UT participants (0.0065 ± 0.0025 vs. 0.0055 ± 0.0019 mm^2^, respectively, *p =* 0.047). There was no effect of biological sex (*p =* 0.344) or training (*p =* 0.484) on fibre length. The MHC isoform of individual fibres was determined using SDS‐PAGE (Figure 2). Fibre‐type distribution differed between males and females such that females exhibited a greater proportion of MHC I fibres than males (28% vs. 6%, respectively). In both groups, MHC II (including IIA, IIX and A/X) fibres composed the majority of the sample (72% in females, 94% in males). Because MHC IIA fibres were the most abundant and were represented similarly across all groups, all statistical analyses for single‐fibre mechanics, both active tension and passive measures, included only MHC IIA fibres, as mentioned above.

**TABLE 2:** Summary statistics of fibres analysed.

Group	Sex	*n*	CSA (mm^2^)^*†^	Length (mm)	Relative % MHC			
					I	IIA	IIX	IIA/X
Untrained	Female	107	0.0046 ± 0.0014	1.5 ± 0.4	22	47	2	22
	Male	92	0.0066 ± 0.0019	1.7 ± 0.4	3	49	23	24
Trained	Female	128	0.0053 ± 0.0016	1.6 ± 0.6	34	47	2	12
	Male	97	0.0081 ± 0.0026	1.8 ± 0.5	9	55	2	34

*Note*: Data are shown as the mean ± SD. Percentages are not reported for MHC I/IIA or I/IIA/IIX because collectively they made up only 3.1% of the overall dataset. Symbols indicate main effects of biological sex (^*^) or training (^†^) (all *p <* 0.05).

To ensure that stress decay was largely complete after each applied strain, the difference between the measured stress and predicted stress, termed ‘missed stress’, was calculated at each stretch step, for each fibre, as described previously (Privett et al., 2024). Stress decay was greatest at SL = 3.2–3.8 µm, suggesting the greatest potential for incomplete stress decay. At these lengths, however, measured stress was different from predicted stress by only 0.08%–2.37%. Given this minimal variation, it is not likely that incomplete stress decay impacted the ability to draw meaningful conclusions from final measured stress, thus measured stress was used throughout.

### 3.4 Maximal isometric tension

**FIGURE 3:**
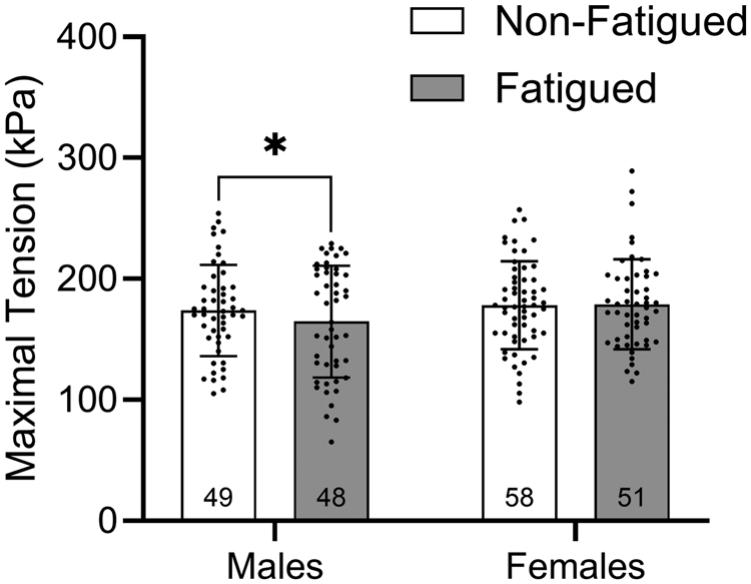
Active tension generation was assessed in myosin heavy chain IIA fibres. There was no significant effect of training, biological sex or fatigue condition on active tension. Active tension was significantly reduced in fatigued fibres of males but not females. Data are shown as the mean ± SD.

Considering the known impact of MHC isoform on active tension and the relative balance in the proportion of MHC IIA fibres across groups (Table 2), maximally active isometric tension was analysed in MHC IIA fibres only. There was no main effect of training (*p =* 0.219), biological sex (*p =* 0.843) or fatigue conditions (*p =* 0.653) on maximal isometric tension; however, the interaction between biological sex and fatigue was significant (*p =* 0.013) such that maximal tension was modestly reduced (∼5%) by fatiguing exercise in males (174.0 ± 37.7 vs. 164.7 ± 46.2 kPa, respectively *p =* 0.021) but not in females (178.1 ± 36.4 vs. 179.0 ± 37.1 kPa, respectively, *p =* 0.163; Figure 3).

### 3.5 Passive stress

**FIGURE 4:**
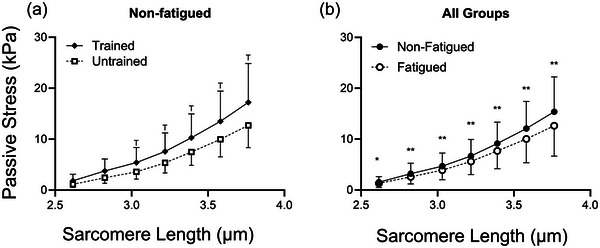
(a) Passive stress in control fibres was significantly greater in trained compared with untrained participants at sarcomere lengths > 3.0 µm; main effect of training (^T^ *p <* 0.05). (b) The collective mean from all participants (male/female, trained/untrained) reveals that passive stress was significantly reduced at all sarcomere lengths with fatigue (effect of fatigue: ^*^ *p <* 0.05 or ^**^ *p <* 0.01). Data are the mean ± SD.

**FIGURE 5:**
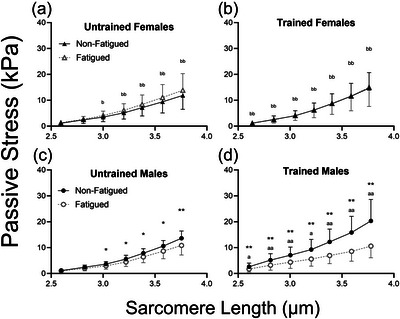
(a,c) In untrained participants, passive stress was significantly reduced by fatiguing exercise at a sarcomere length > 3.0 µm in males but not in females (c; fatigue response different from that of males: ^b^ *p <* 0.05 or ^bb^ *p <* 0.01). (b,d) In trained male and female participants, the effect of fatigue on passive stress was highly significant at all sarcomere lengths in males (d) but not in females (b) [note that the means of fatigued and non‐fatigued fibre passive stress are sufficiently close in (b) that the data points obscure one another] with highly significant fatigue by sex interactions in females (fatigue response different from that of males: ^b^ *p <* 0.05 or ^bb^ *p <* 0.01). Fatigue response was affected by training such that trained males experienced greater fatigue‐related deficits than untrained males (fatigue response different from that of untrained: ^a^ *p <* 0.05 or ^aa^ *p <* 0.01).

When considering only non‐fatigued fibres (Figure 4a), passive stress was significantly higher in RT versus UT fibres at longer SL (*p =* 0.033 at SL 3.0 µm, *p =* 0.035 at SL 3.2 µm, *p =* 0.034 at SL 3.4 µm, *p =* 0.032 at SL 3.6 µm and *p =* 0.034 at SL 3.8 µm). However, there was no main effect of biological sex on passive stress in non‐fatigued fibres, at any SL. Testing the effect of fatigue across all groups revealed that passive stress was significantly reduced in fatigued fibres (*p =* 0.034 at SL = 2.6 µm and *p <* 0.01 at SL = 2.8–3.8 µm; Figure 4b). Fatigue by training interactions were observed at multiple SL (*p =* 0.049 at SL 2.6 µm, *p =* 0.038 at SL 2.8 µm, *p =* 0.028 at SL 3.0 µm, *p =* 0.023 at SL 3.2 µm, *p =* 0.017 at SL 3.4 µm, *p =* 0.013 at SL 3.6 µm and *p =* 0.017 at SL 3.8 µm), and fatigue by sex interactions were observed at all SL (*p <* 0.01) such that fatigue reduced passive stress more in males than females. Therefore, additional analyses were performed to compare the impact of fatigue in each group (Figure 5a–d). In UT females (Figure 5a), passive stress was not significantly different according to fatigue at any SL. Furthermore, in fibres from UT participants, the effect of fatiguing exercise on passive stress differed by biological sex at longer SL (*p =* 0.019 at SL 3.0 µm and *p <* 0.01 at SL 3.2–3.8 µm). In UT males (Figure 5c), passive stress was significantly lower in fatigued versus non‐fatigued fibres at longer lengths (*p =* 0.032 at SL 3.0 µm, *p =* 0.018 at SL 3.2 µm, *p =* 0.018 at SL 3.4 µm, *p =* 0.010 at SL 3.6 µm and *p <* 0.01 at SL 3.8 µm). In RT males (Figure 5d), passive stress was significantly reduced by fatigue at all SL (*p <* 0.01 at SL 2.6–3.8 µm), and the response of passive stress to fatigue was greater than that of UT males (interaction *p =* 0.018 at SL 2.6 µm, *p <* 0.01 at SL 2.8–3.0 µm, *p =* 0.010 at SL 3.2 µm and *p <* 0.01 at SL 3.4–3.8 µm). In fibres from RT females (Figure 5b), fatigue did not significantly affect passive stress at any SL, and the response of passive stress to fatiguing exercise was different from that of RT males at all SL (*p <* 0.01 at SL 2.6–3.8 µm).

### 3.6 Passive Young's modulus

#### 3.6.1 Short lengths

**FIGURE 6:**
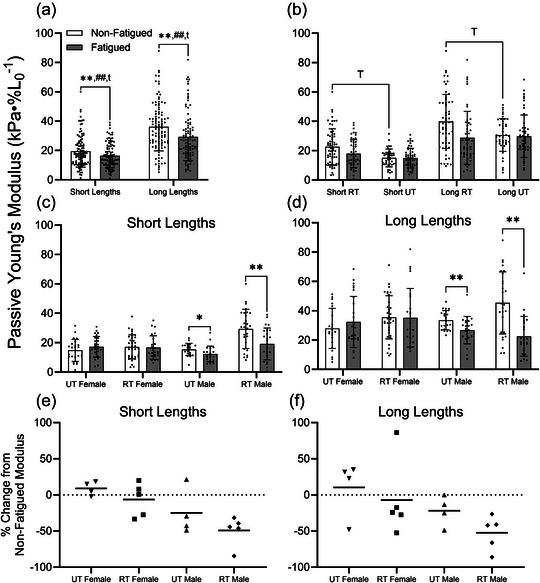
(a) Passive Young's modulus was significantly reduced by fatiguing exercise at short and long lengths. Furthermore, the biological sex by fatigue and training by fatigue interactions were significant at short and long lengths. (b) In non‐fatigued fibres, passive Young's modulus was significantly greater in RT versus UT individuals at short and long lengths. (c,d) At short (c) and long (d) sarcomere length, it becomes evident that reductions in passive modulus were driven by RT and UT males, but not females. (e,f) On a per‐individual basis, passive modulus was reduced in 9 of the 10 included males at short (e) and long (f) lengths. However, females exhibited little to no change in passive modulus at short lengths and varied responses at long lengths. Data represent the mean ± SD. Symbols indicate a significant effect of fatigue (^*^ *p <* 0.05 or ^**^ *p *< 0.01), fatigue by training interaction (^t^ *p *< 0.05), fatigue by biological sex interaction (^##^ *p *< 0.01) or main effect of training (^T^ *p *< 0.05). Abbreviations: RT, resistance trained; UT, untrained.

Analyses of the passive modulus included only MHC IIA fibres. At short fibre lengths, the passive modulus was significantly reduced by fatigue (19.5 ± 10.8 vs. 16.4 ± 8.3 kPa %*L*_0_^−1^, respectively, *p =* 0.006; Figure 6a). The significant interaction between fatigue and training (*p =* 0.044) prompted assessment of the effect of training on the passive modulus at short lengths in non‐fatigued and fatigued fibres, separately. As a result, the passive modulus was significantly higher in RT versus UT non‐fatigued fibres (22.6 ± 12.3 vs. 15.1 ± 6.1 kPa %*L*_0_^−1^, respectively, *p =* 0.031) but not fatigued fibres (*p =* 0.406; Figure 6b). Additionally, the interaction between fatigue and biological sex (*p <* 0.01) was statistically significant (Figure 5a), prompting *post hoc* analysis via separate mixed models for males and females. These follow‐up analyses (Figure 6c) revealed that the passive modulus was significantly reduced by fatigue in males (23.4 ± 12.5 vs. 15.8 ± 9.2 kPa %*L*_0_^−1^, respectively, *p <* 0.001) but not in females (16.2 ± 7.9 vs. 16.9 ± 7.4 kPa %*L*_0_^−1^, respectively, *p =* 0.380). A significant fatigue by training interaction (*p =* 0.022) in the male cohort prompted analysis of the fatigue effect in RT and UT males, separately. The passive modulus was significantly reduced by fatiguing exercise in fibres from RT (29.4 ± 13.3 vs. 19.1 ± 10.8 kPa %*L*_0_^−1^, respectively, *p <* 0.001) and UT (15.4 ± 4.2 vs. 12.3 ± 5.3 kPa %*L*_0_^−1^, respectively, *p =* 0.024) males at short lengths.

#### 3.6.2 Long lengths

At long fibre lengths, the passive modulus was significantly reduced by fatigue (36.1 ± 16.4 vs. 29.3 ± 16.2 kPa %*L*_0_^−1^, respectively, *p =* 0.004; Figure 6a) and was significantly altered by interactions between biological sex and condition (*p <* 0.001) and between training and condition (*p =* 0.037). Subsequent analyses revealed that training significantly increased the passive modulus of non‐fatigued fibres (39.9 ± 18.4 vs. 30.6 ± 11.1 kPa %*L*_0_^−1^, respectively, *p =* 0.045) but not fatigued fibres (*p =* 0.925; Figure 6b). Additionally, fatigue reduced the passive modulus of fibres from males (40.3 ± 17.4 vs. 24.6 ± 11.8 kPa %*L*_0_^−1^, respectively, *p <* 0.001) but not females (*p =* 0.296). Furthermore, the significant training by condition interaction in the male cohort revealed that the passive modulus was significantly reduced by fatigue in RT males (45.4 ± 21.2 vs. 22.6 ± 13.6 kPa %*L*_0_^−1^, respectively, *p <* 0.001) and UT males (33.6 ± 6.5 vs. 26.8 ± 9.3 kPa %*L*_0_^−1^, respectively, *p =* 0.004) at long lengths (Figure 6d).

Figure 6e,f illustrates the individual responses, expressed as a percentage difference between the passive modulus of non‐fatigued fibres and that of fatigued fibres. Although RT and UT males demonstrated a reduced passive cellular modulus in fatigued versus non‐fatigued fibres at short (Figure 6e) and long (Figure 6f) lengths, the response in females varied considerably by individual, especially at long lengths.

### 3.7 Stress decay index

**FIGURE 7:**
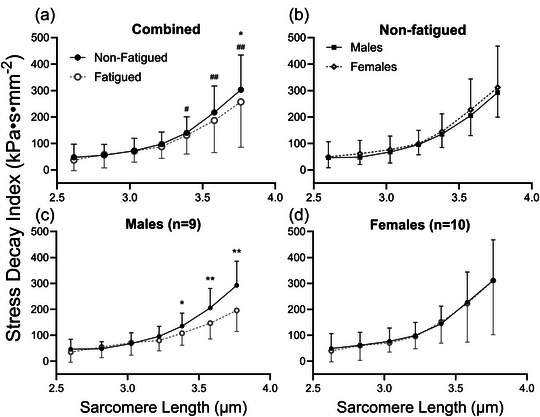
(a) In the combined dataset, neither biological sex nor training status had a significant main effect on SDI at any SL. However, the biological sex by fatigue interaction was significant at SL = 3.4–3.8 µm, and fatigue significantly reduced SDI at SL = 3.8 µm. (b) In non‐fatigued fibres, there was no difference in SDI between males and females, at any SL. (c) The SDI was significantly reduced by fatiguing exercise at SL = 3.4–3.8 µm. (d) On the contrary, females did not exhibit changes in SDI resulting from fatiguing exercise at any SL. Data are shown as the mean ± SD. Symbols indicate a significant effect of fatigue (^*^ *p <* 0.05 or ^**^ *p <* 0.01) or fatigue by biological sex interaction (^#^ *p <* 0.05 or ^##^ *p <* 0.01). Abbreviations: SDI, stress decay index; SL, sarcomere length.

There were no significant effects of biological sex (*p =* 0.657 at SL 2.6 µm, *p =* 0.437 at SL 2.8 µm, *p =* 0.968 at SL 3.0 µm, *p =* 0.530 at SL 3.2 µm, *p =* 0.193 at SL 3.4 µm, *p =* 0.419 at SL 3.6 µm and *p =* 0.435 at SL 3.8 µm) or training (*p =* 0.452 at SL 2.6 µm, *p =* 0.500 at SL 2.8 µm, *p =* 0.752 at SL 3.0 µm, *p =* 0.429 at SL 3.2 µm, *p =* 0.269 at SL 3.4 µm, *p =* 0.706 at SL 3.6 µm and *p =* 0.945 at SL 3.8 µm) on SDI at any SL. In contrast, SDI was significantly reduced in fatigued versus non‐fatigued fibres at SL 3.8 µm only (257.43 ± 171.75 vs. 303.08 ± 131.20 kPa s mm^−2^, *p =* 0.034) but not at any other length (*p =* 0.079 at SL 2.6 µm, *p =* 0.656 at SL 2.8 µm, *p =* 0.704 at SL 3.0 µm, *p =* 0.151 at SL 3.2 µm, *p =* 0.449 at SL 3.4 µm and *p =* 0.074 at SL 3.6 µm; Figure 7a). Furthermore, there were significant sex by fatigue interactions at SL = 3.4 µm (*p =* 0.021), 3.6 µm (*p =* 0.007) and 3.8 µm (*p =* 0.001) but not at other SLs (*p =* 0.871 at SL 2.6 µm, *p =* 0.476 at SL 2.8 µm, *p =* 0.188 at SL 3.0 µm and *p =* 0.329 at SL 3.2 µm). As a result, the effect of fatigue on SDI was examined in males and females, separately, at these SLs. In non‐fatigued fibres only, there were no differences in SDI between males and females at any SL (*p =* 0.804 at SL 2.6 µm, *p =* 0.180 at SL 2.8 µm, *p =* 0.424 at SL 3.0 µm, *p =* 0.818 at SL 3.2 µm, *p =* 0.640 at SL 3.4 µm, *p =* 0.740 at SL 3.6 µm and *p =* 0.902 at SL 3.8 µm; Figure 7b). In males, SDI was significantly reduced in fatigued versus non‐fatigued fibres at SL = 3.2 µm (96.17 ± 38.49 vs. 80.13 ± 39.91 kPa s mm^−2^, respectively, *p =* 0.027), SL = 3.4 µm (108.00 ± 46.54 vs. 135.27 ± 50.31 kPa s mm^−2^, respectively, *p =* 0.002), 3.6 µm (147.08 ± 61.32 vs. 205.33 ± 75.27 kPa s mm^−2^, respectively, *p <* 0.001) and 3.8 µm (195.78 ± 81.11 vs. 292.59 ± 92.99 kPa s mm^−2^, respectively, *p <* 0.001), but not at any other SLs (*p =* 0.133 at SL 2.6 µm, *p =* 0.257 at SL 2.8 µm and *p =* 0.189 at SL 3.0 µm; Figure 7c). However, fatigue had no effect on SDI at any SL in fibres from females (*p =* 0.326 at SL 2.6 µm, *p =* 0.781 at SL 2.8 µm, *p =* 0.469 at SL 3.0 µm, *p =* 0.698 at SL 3.2 µm, *p =* 0.394 at SL 3.4 µm, *p =* 0.564 at SL 3.6 µm and *p =* 0.482 at SL 3.8 µm; Figure 7d).

**FIGURE 8:**
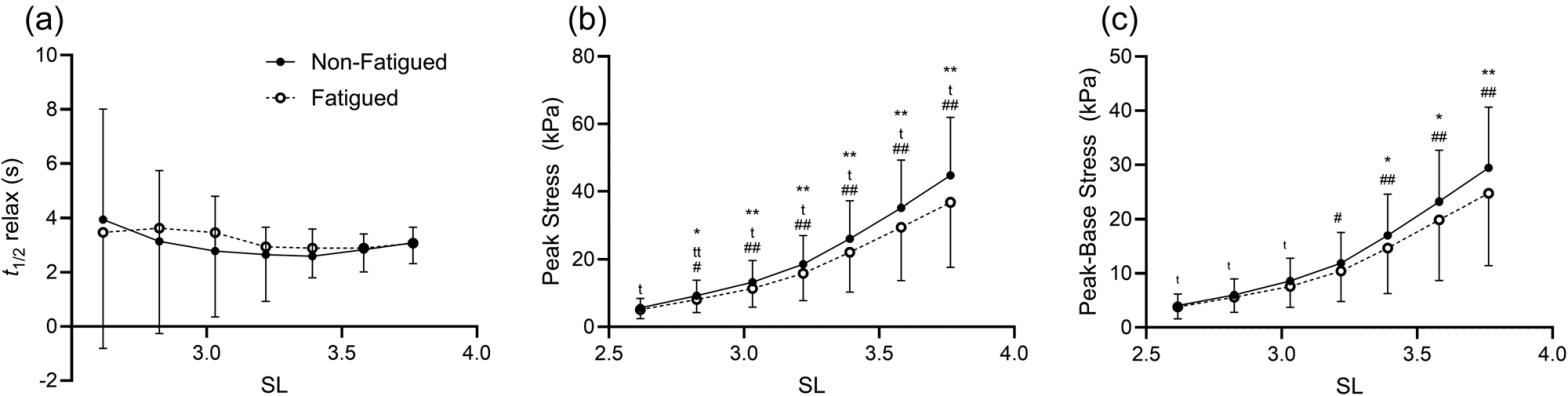
(a) When considering the measures used to calculate the stress decay index, it becomes clear that half‐relaxation time is not impacted by fatiguing exercise. (b,c) However, both peak stress (b) and the magnitude of stress decay (c) are reduced by fatiguing exercise, with both measures demonstrating mediating effects of biological sex and training status on the response to fatigue. Data are shown as the mean ± SD. Symbols indicate a significant effect of fatigue (^*^ *p <* 0.05 or ^**^ *p <* 0.01), fatigue by training interaction (^t^ *p <* 0.05 or ^tt^ *p <* 0.01) or fatigue by biological sex interaction (^#^ *p <* 0.05 or ^##^ *p <* 0.01). Abbreviations: SL, sarcomere length, *t*_½_ relax, half‐relaxation time.

To consider the underlying contributors to the fatigue‐induced shift in SDI, we examined the measures that were used to calculate SDI: half‐relaxation time, peak stress and the magnitude of stress decay (peak stress minus baseline stress). There were no significant differences in half‐relaxation time attributable to fatiguing exercise, biological sex or training at any SL (all *p <* 0.05; Figure 8a). However, both peak stress and the magnitude of stress decay were significantly different according to fatigue, with significant interactions of biological sex by fatigue and training by fatigue. Specifically, peak stress was significantly different by fatigue (*p =* 0.026 at SL = 2.8 µm, *p* ≤ 0.01 at SL = 3.0–3.8 µm), fatigue by training interactions (*p <* 0.01at SL 2.6, *p =* 0.012 at SL 2.8 µm, *p =* 0.012 at SL 3.0 µm, *p =* 0.033 at SL 3.2 µm, *p =* 0.049 at SL 3.4 µm, *p =* 0.047 at SL 3.6 µm and *p =* 0.026 at SL 3.8 µm) and fatigue by sex interactions (*p =* 0.022 at SL = 2.8 µm and *p <* 0.01 at SL 3.0–3.8 µm; Figure 8b). Finally, the magnitude of stress decay was impacted by fatigue (*p =* 0.030 at SL 3.4 µm, *p =* 0.014 at SL 3.6 µm and *p <* 0.01 at SL 3.8 µm) and the interaction of biological sex by fatigue (*p =* 0.028 at SL 3.2 µm and *p <* 0.01 at SL 3.4–3.8 µm) at long lengths and by the interaction of training by fatigue at short lengths (*p =* 0.023 at SL 2.6 µm, *p =* 0.013 at SL 2.8 µm and *p =* 0.042 at SL 3.0 µm; Figure 8c).

**FIGURE 9:**
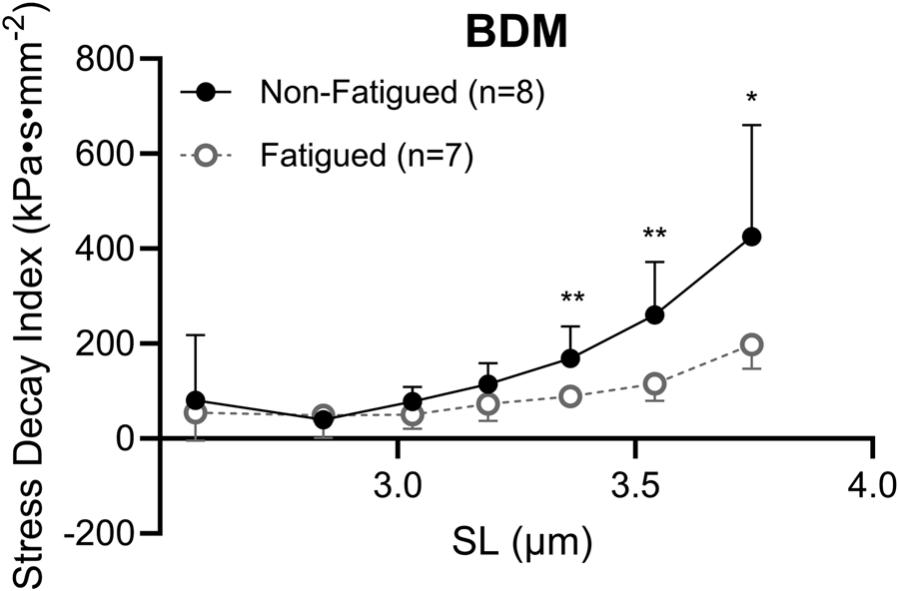
In BDM‐treated fibres from two untrained males, fatigue‐induced differences in the stress decay index persisted at SL 3.4–3.8 µm, suggesting that intracellular proteins other than myosin and actin contribute to this phenomenon. Data represent the mean ± SD. Symbols indicate a significant effect of fatigue (^*^ *p <* 0.05 or ^**^ *p <* 0.01). Abbreviations: BDM, 2,3‐butanedione monoxime; SL, sarcomere length.

To test whether spontaneous actomyosin interactions while in relaxing solution (pCa 8.0) contributed to observed differences in SDI, fatigue‐induced differences in SDI were assessed in a subset of fibres from two UT young males treated with 40 mM BDM. In these BDM‐treated fibres, SDI was significantly reduced in fatigued compared with non‐fatigued fibres at SL = 3.4 µm (89.06 ± 19.52 vs. 169.16 ± 66.95 kPa s mm^−2^, respectively, *P =* 0.009), 3.6 µm (115.40 ± 35.55 vs. 260.22 ± 112.20 kPa s mm^−2^, respectively, *p =* 0.006) and 3.8 µm (197.54 ± 50.78 vs. 425.61 ± 234.63 kPa s mm^−2^, respectively, *p =* 0.026; Figure 9), consistent with observations in non‐treated fibres.

### 3.8 Tissue‐level measures

**FIGURE 10:**
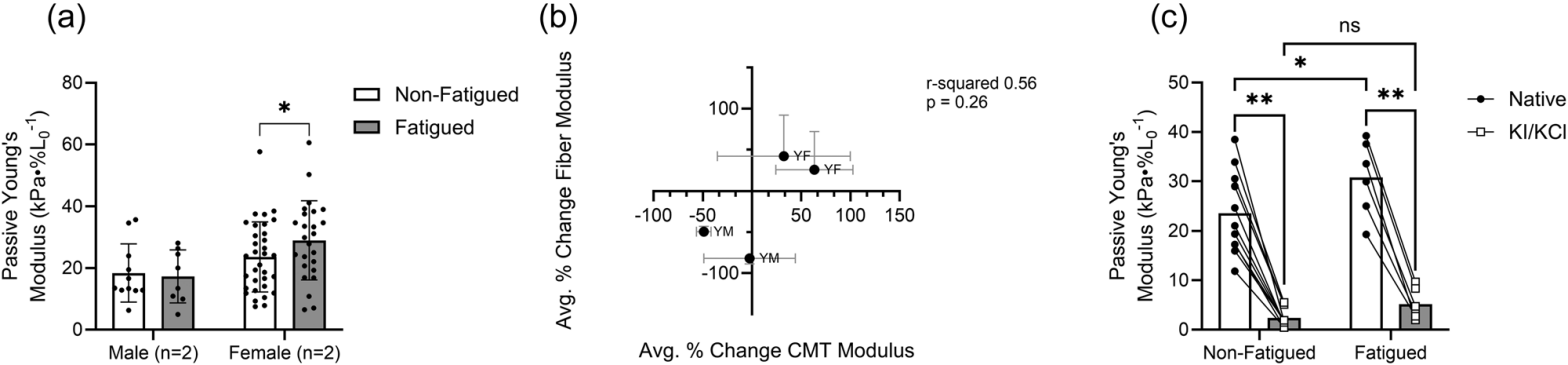
(a) Although the tissue‐level passive modulus was not affected by fatiguing exercise in the male cohort, it was significantly increased by fatiguing exercise in the female cohort (*p =* 0.036). (b) The direction of change in the passive modulus was similar between fibres and CMT for each participant; the correlation of change was insignificant. (c) Fatigue‐induced differences in passive modulus were detected at the level of CMT; however, chemical elimination of intracellular contributors to passive modulus abolished the fatigue effect. The passive modulus was likewise reduced by treatment in fatigued and non‐fatigued CMT. Each point represents one CMT sample, with lines connecting the pre‐ and post‐treatment values. Symbols indicate fatigue difference (^*^ *p <* 0.05 or ^**^ *p <* 0.001). Abbreviation: CMT, composite muscle tissue.

Owing to the linearity of the stress–strain curve for tissue samples, the passive Young's modulus was quantified as the slope of the entire stress–strain curve. When the cohort was studied as a whole, there was no significant difference between modulus values of males and females (14.34 ± 9.67 vs. 25.91 ± 12.22 kPa %*L*_0_^−1^, respectively, *p =* 0.197) or of non‐fatigued and fatigued samples (21.06 ± 11.67 vs. 23.93 ± 13.76 kPa %*L*_0_^−1^, respectively, *p =* 0.287). However, given the relatively limited sample in males versus females (26 vs. 58 bundles, respectively), a subsequent analysis tested the effect of fatigue condition on tissue passive modulus in females only. This sample was limited *post hoc* by our desire to match bundle size across samples. In order to account for the potential effect of bundle size on passive modulus (Malakoutian et al., 2021), we eliminated bundles from analyses that were 2SD below or above or below the inclusive mean for CSA (*n* = 7). Our restricted analysis revealed a modest but significant increase in the passive modulus of the fatigued versus non‐fatigued bundles (28.98 ± 12.85 vs. 23.57 ± 11.36 kPa %*L*_0_^−1^, *p =* 0.036) in the female cohort but no difference in the males (non‐fatigued, 18.37 ± 9.44 kPa %*L*_0_^−1^; fatigued, 17.28 ± 8.62 kPa %*L*_0_^−1^, *p =* 0.801; Figure 10a). Notably, a similar result was produced when a wider range of tissue sizes were analysed (data not shown).

To assess whether cellular changes in the passive modulus resulting from fatiguing exercise were associated with the same changes in the CMT modulus, the average change in fibre versus CMT passive modulus was plotted for each participant for whom both types of sample were tested. Although values were clustered by biological sex, and changes in fibre and CMT values with fatigue were relatively consistent, the correlation between change in fibre modulus versus that of CMT was not significant (*p =* 0.26; Figure 10b).

Finally, passive modulus measures from CMT samples of one RT female were collected before and after incubation in KI and KCl. Before treatment, the passive modulus was significantly higher in fatigued versus non‐fatigued CMT (30.78 ± 7.62 vs. 23.59 ± 8.39 kPa %*L*_0_^−1^, respectively, *p =* 0.025; Figure 10c). However, the passive modulus following KI/KCl treatment was not different between fatigued (5.17 ± 3.13 kPa %*L*_0_^−1^) and non‐fatigued (2.40 ± 1.88 kPa %*L*_0_^−1^) CMT (*p =* 0.370). As anticipated, KI/KCl treatment significantly and dramatically reduced the passive modulus of non‐fatigued and fatigued fibres (*p <* 0.001 for both).

## 4 DISCUSSION

### 4.1 Overall

Our study sought to further our prior investigations that revealed sex‐based differences in the acute response to fatigue such that males demonstrated reductions in the myocellular modulus in response to fatiguing exercise, but females did not. In the present study, we recruited additional individuals, including age‐matched males and females who engaged in frequent, high‐intensity exercise to test whether chronic physical exercise would manifest enhanced myofibre modulus in humans and whether the sex‐based variation in the acute response to fatigue was modified by chronic effectors of modulus. We report persistent responsiveness to acute fatigue in males that was not present in either trained or sedentary female groups. Indeed, chronic physical training enhanced modulus regardless of sex, whereas acute responses were absent in females, reinforcing our previous findings and providing additional insights discussed below.

### 4.2 Study sample

In the present group of participants, height, weight and body mass index were greater in males than females (Table 1). By design, physical activity as measured by step count, and time spent in moderate activity per day was higher in the RT compared with UT participants (Table 1). The impact of this difference in activity was evident in whole‐muscle performance measures (Table 3) and fibre CSA, all of which were higher in RT versus UT participants. However, there was no effect of biological sex or training on the duration of fatiguing exercise or the fatigue ratio, an index of the magnitude of fatigue (Table 3). Thus, it is unlikely that interactions between fatigue and biological sex or training are attributable to differences in the duration of fatiguing exercise or magnitude of in vivo fatigue. Finally, although fibre‐type distribution and fibre CSA differed across training and biological sex groups (Table 2), final analyses included only MHC IIA fibres and mechanics measures were reported normalized to CSA to minimize confounding effects on outcome measures. Although restricting assessment to MHC IIA fibres somewhat limits our ability to make more broad generalizations about the applicability of our findings, we remain confident that the phenomenon of fatigue‐induced changes in muscle tissue mechanics reported here persists across multiple fibre types. Indeed, in separate analyses including all measured fibres, regardless of fibre type (*n* = 423), conclusions related to the effect of fatiguing exercise on passive stress and Young's modulus were not altered. However, variations by fibre type across conditions contributed significant variability that disrupted observed interactions between main effects of fatigue and training/sex. Therefore, we sought to avoid the potential for confounding interpretations, based on different fibre‐type distributions across groups, by limiting our assessments to samples where the MHC isoform was relatively equally represented across conditions (MHC IIA; see Table 2).

**TABLE 3:** Fatigue data and whole‐muscle performance.

Group	Sex	Time to fatigue (s)	Fatigue ratio	Peak power^*†^ (W)	Relative peak power^*†^ (W kg^−1^)	Peak torque^*†^ (N m)	Relative peak torque^*†^ (N kg^*†^)
Untrained	Female	68 ± 8.8	0.34 ± 0.10	295 ± 33	5.43 ± 0.32	140 ± 8	2.54 ± 0.12
	Male	75 ± 29	0.41 ± 0.16	532 ± 95	6.43 ± 1.42	281 ± 44	3.34 ± 0.34
Trained	Female	99 ± 74	0.36 ± 0.12	383 ± 73	6.19 ± 0.68	195 ± 62	3.20 ± 0.73
	Male	71 ± 14	0.28 ± 0.10	834 ± 252	10.37 ± 1.85	364 ± 44	4.62 ± 0.53

*Note*: Data are shown as the mean ± SD. Symbols indicate a significant main effect of biological sex (^*^)or training (^†^) between groups (*p <* 0.05).

### 4.3 Cellular active mechanics

Active tension was not significantly different by biological sex or by training group. However, a significant fatigue by biological sex interaction revealed a modest yet significant reduction in active tension of fatigued versus non‐fatigued fibres in males only (Figure 3). This has been observed previously (Privett et al., 2021; Ricci et al., 2023) and might be attributable to exercise‐induced PTM of sarcomere proteins involved in active contractile function. For example, slow skeletal myosin binding protein‐C (ssMyBP‐C), a thick filament regulatory protein that interacts with both the thick and thin filaments to regulate crossbridge formation (Korte et al., 2003; Robinett et al., 2019; Yamamoto, 1986), is phosphorylated by fatiguing exercise (Privett et al., 2021; Ricci et al., 2023). Dephosphorylated ssMyBP‐C is more likely to bind to both the thick and thin filaments, resulting in slowed crossbridge kinetics, whereas phosphorylation releases this clutch‐like mechanism, enhancing crossbridge kinetics (Colson, 2019; Robinett et al., 2019). Therefore, it is plausible that accelerated crossbridge kinetics resulting from ssMyBP‐C phosphorylation during fatiguing exercise might reduce isometric tension. Although we can only speculate regarding the mechanisms explaining this phenomenon, tension production in these fibres clearly demonstrates adequate function of all fibres included in the present analyses and supports the notion that structural damage was not present in these fibres.

### 4.4 Chronic training increases baseline passive stress and Young's modulus at longer lengths

In non‐fatigued fibres, passive stress (Figure 4a) and Young's modulus (Figure 6d) were higher in fibres from RT versus UT participants at longer SLs. This is the first observation of training‐related differences in the passive modulus in humans, a phenomenon previously observed in rodents (Noonan et al., 2020). Although chronic training might impact ECM‐based stiffness in skeletal muscle (Kjær, 2004), this is unlikely to influence the cellular mechanics reported in the present study owing to the use of chemically permeabilized skeletal muscle fibres, which no longer have intact ECM. Instead, it is more likely that intracellular proteins are responsible for the observed training effect on passive measures. The fact that training effects were observed at longer SLs (3.0–3.8 µm), where overlap of thick and thin filaments is minimal, suggests that the sarcomere protein titin plays a significant role.

Differential expression of titin isoforms across participants has been reported previously (Fry et al., 1997), presenting the possibility that training‐induced shifts in titin isoform distribution might have contributed to the observed increase in passive stress or modulus in RT versus UT participants. Yet, more recent studies cast doubt on the notion that training‐induced shifts in titin isoform (McGuigan et al., 2003; Pellegrino et al., 2016) or titin size (Noonan et al., 2020) explain variations in skeletal muscle mechanics. Nonetheless, 3 weeks of exercise training has been demonstrated to shift the titin isoform in murine cardiac muscle (Hidalgo et al., 2014), suggesting that, although the effect of exercise on titin isoform might be specific to training modality and muscle type, isoform shifts are possible in striated muscle. It is also possible that chronic training alters titin‐based stiffness through changes to titin PTMs. Training‐based differences in titin phosphorylation, a well‐studied mediator of titin‐based stiffness, have been observed in murine cardiac muscle (Hidalgo et al., 2014). However, it is not yet known whether chronic training alters the phosphorylation background of titin in human skeletal muscle, and we did not report titin phosphorylation measures in the present study. Although it is unclear how chronic training impacts skeletal muscle titin in humans specifically, there is certainly a role for titin in skeletal muscle mechano‐signalling and remodelling in response to exercise (Krüger & Kötter, 2016), supporting the notion that titin‐based stiffness might adapt to a chronic training stimulus in order to meet the demands of intense physical exercise better.

### 4.5 Fatiguing exercise alters cellular passive mechanics

In support of our initial hypothesis, fatiguing exercise significantly reduced both passive stress (Figures 4b) and Young's modulus (Figure 6a) when participants were considered collectively. Given that the effect of fatiguing exercise on passive stress and modulus was mediated by biological sex and training status, subsequent analyses examined training‐ and biological sex‐based groups separately. As a result, it became clear that trained males had the most robust reduction in passive stress (Figure 5d) and modulus (Figure 6c,d) in fatigued versus non‐fatigued fibres, yet untrained females exhibited a non‐significant upward trend in passive stress (Figure 5a) and modulus (Figure 6d) in fatigued versus non‐fatigued fibres. In all groups where a mean difference (even a non‐significant one) in passive stress or modulus was observed between non‐fatigued and fatigued fibres, the difference increased as SL increased. In fact, the difference in mean passive stress between fatigued and non‐fatigued fibres was largest at longer SLs (∼3.4 µm and longer), where thick and thin filaments no longer overlap. Although the contribution of residual actomyosin interactions to cellular passive mechanical properties is possible, we have previously demonstrated that cellular passive modulus is still altered by fatiguing exercise in fibres treated with BDM, a myosin inhibitor, suggesting that residual crossbridge formation is not a primary mechanism of fatigue‐induced changes to cellular passive mechanics (Privett et al., 2024). Rather, the fact that fatigue‐induced differences are largest at longest lengths, where passive mechanics in permeabilized muscle fibres are predominantly titin based, suggests that titin‐based mechanisms might have contributed to these observations. Titin‐based stiffness can be regulated acutely via PTM, and it is possible that exercise‐induced small molecules, such as inorganic phosphate (Hamdani et al., 2017; Müller et al., 2014), oxidative molecules (Alegre‐Cebollada et al., 2014) or heat shock proteins (Kötter et al., 2014), bind to viscoelastic regions of titin during fatiguing exercise, thereby altering titin‐based viscoelasticity.

We have previously reported altered titin phosphorylation following a single bout of fatiguing exercise (Privett et al., 2024), which might have implications for titin‐based stiffness (Hamdani et al., 2017). However, the specific response of titin‐based stiffness to phosphorylation is highly dependent on the location at which the phosphorylation event occurs, as has been observed previously (Müller et al., 2014). Separately, *S*‐glutathionylation of cryptic binding sites of cardiac titin has been shown to inhibit titin immunoglobulin domain refolding, thereby reducing titin‐based stiffness (Alegre‐Cebollada et al., 2014). In skeletal muscle, *S*‐glutathionylation has been demonstrated to reduce passive force resulting from fibre stretch, yet the observed reductions were smaller than those observed in cardiac muscle (Watanabe et al., 2020). However, oxidation events, such as *S*‐glutathionylation, are not likely to be present in our sample owing to the use of the antioxidant DTT in dissecting and relaxing solutions. Finally, it is possible that small HSPs generated during fatiguing exercise might bind to titin in a way that alters titin‐based stiffness. Previous work reported the translocation of small HSPs, αβ‐crystallin and HSP27, to the elastic regions of skeletal muscle titin (Kotter et al., 2014). In the same study, the binding of these small HSPs in cardiac myocytes prevented acidification‐based aggregation of titin immunoglobulin domains and subsequent increases in titin‐based stiffness. In the present study, we did not measure changes to titin PTMs, precluding the ability to draw conclusions regarding their contribution to the observed changes in passive stress and modulus. However, these passive measures were made in permeabilized single skeletal muscle fibres, suggesting that the observed differences were attributable, at least in part, to a titin‐based mechanism. Further studies in this area should seek to determine where exercise‐induced changes to titin phosphorylation occur along the titin protein (Privett et al., 2024) and should assess whether the binding of small HSPs to the elastic regions of skeletal muscle titin is altered by an acute bout of fatiguing exercise.

### 4.6 Biological sex and chronic training mediate the effect of fatiguing exercise on cellular passive stress and Young's modulus

Neither the duration of fatiguing exercise nor the fatigue ratio differed by biological sex or training status; therefore, the sex‐ and training‐based differences in response to fatiguing exercise are not likely to be explained by differences in performance of the fatiguing exercise itself (Table 3). Rather, the lack of a significant mean response in females was attributable, at least in part, to the variability in response to fatigue among female participants (Figure 6e), especially at long lengths (Figure 6f). One potential explanation for this varied response might be sex‐based differences in circulating levels of oestrogen (Bell et al., 2012, 2011; Ham et al., 2020). Specifically, despite our efforts to minimize variability by collecting biopsy samples from the same point of the menstrual cycle (prefollicular phase, *n* = 5) or from participants using hormonal contraception (*n* = 5), inter‐individual differences in systemic oestrogen might have contributed to differences in skeletal muscle mitochondrial function (Pellegrino et al., 2022; Yoh et al., 2023). Mitochondrial function can affect the concentration of heat shock proteins (Liu & Steinacker, 2001) and oxidative molecules, such as glutathione (Marí et al., 2009), both of which have the capacity to alter titin‐based stiffness via PTM, in the sarcoplasm. Given the potential impact of varied blood oestrogen concentration on intracellular mediators of skeletal muscle stiffness, future studies will seek to measure circulating oestrogen concentration at the time of muscle biopsy.

The response of cellular passive mechanics was also mediated by training status. Although training‐induced changes to skeletal muscle titin isoform are possible, there is no evidence for training‐induced shifts in skeletal muscle titin in the literature (McGuigan et al., 2003; Pellegrino et al., 2016). However, training might modify the pattern of PTMs to titin. There is evidence that titin‐based stiffness can be modified by phosphorylation (Hamdani et al., 2017) and *S*‐glutathionylation (Alegre‐Cebollada et al., 2014), yet there is no direct evidence suggesting that chronic training results in baseline changes to either. HSPs, in contrast, have been demonstrated to increase in baseline expression after training (Morton et al., 2009). Therefore, it is possible that HSPs exert a greater influence in the skeletal muscle of trained versus untrained young adults. For this reason, future studies should seek to measure HSP binding to titin in human VL samples.

### 4.7 Stress decay index

Although the incremental stretch protocol used here precludes the measurement of viscosity, per se, SDI is a useful proxy for assessing the impact of fatiguing exercise on VL viscoelasticity (Lim et al., 2019). In the results reported here, there is a clear mediating effect of biological sex on the response of SDI to fatiguing exercise such that fatigue reduces SDI at longer SL in samples from males but not females (Figure 7). To interrogate the underlying contributors to fatigue‐induced alteration to SDI, half‐relaxation time, peak stress and magnitude of stress decay were analysed (Figure 8). There was no effect of fatigue, biological sex or training on half‐relaxation time, suggesting that these factors did not impact the time course of stress decay. Rather, the peak stress and the magnitude of stress decay were both reduced in fatigued versus non‐fatigued fibres, with mediating effects of training and/or biological sex on fatigue response in a length‐dependent manner. In skeletal muscle myofibrils, viscoelastic behaviour arises from titin immunoglobulin domain unfolding such that the amplitude of force decay increases as SL increases (Minajeva et al., 2001), paralleling the results presented here (Figure 8c). The possibility that residual actomyosin binding interactions in our relaxing solution (pCa 8.0) might have contributed to the observed differences in SDI was considered. However, the effect of fatigue on SDI was still present in BDM‐treated fibres (Figure 9), suggesting that actomyosin binding was unlikely to be a primary contributor to fatigue‐induced differences in SDI. The present study design does not allow us to draw strong conclusions regarding the physiological importance of fatigue‐induced changes to titin viscoelasticity to whole‐muscle function. However, one previous study used a unique knockout model targeting the PEVK region, a prominent contributor to cardiac viscoelasticity, to produce a 40% reduction in left ventricular chamber viscosity in the PEVK knockout hearts (Chung et al., 2011). Although it is important to acknowledge the potential muscle specificity of the mechanisms and physiological relevance of titin‐based viscoelastic properties, this work highlights the potential role of titin in modulating whole‐muscle viscoelasticity. As such, future studies should include methods to measure true viscosity of fatigued and non‐fatigued skeletal muscle cells and composite tissue.

### 4.8 Contribution of intracellular proteins to tissue‐level passive modulus

To explore how altered cellular passive modulus might impact mechanics at the tissue level, the effect of fatiguing exercise on CMT samples was considered in a subset of males and females (Figure 10a). The CMT modulus was not different following fatigue in males but was elevated following fatigue in females. Although seemingly in contrast to fibre‐level data in the more complete sample, this observation was consistent with fibre‐level differences from the four individuals included in this subset. This parallel response in fibre and CMT modulus to fatiguing exercise supported our interpretation that intracellular mediators of passive modulus contribute to passive mechanics at higher levels of organization, although we were unable to assess their relative contribution directly from the data presented here (Figure 10b). These associations were not significant and are interpreted with some caution, given prior evidence that the inclusion of ECM (Mathewson et al., 2014; Ward et al., 2020) might obscure the influences of the myofibre.

CMT samples from one RT female were subjected to passive stretch before and after treatment with KI and KCl to eliminate sarcomere thin and thick filaments, respectively. The removal of thick and thin filaments via KI/KCl is an established method (Ottenheijm et al., 2012) to eliminate titin‐based contributions to cardiac and skeletal muscle stiffness. Therefore, any remaining passive force measured after treatment is attributed to ECM. In the subset of CMT samples used for KI/KCl experiments, CMT passive modulus was higher in fatigued versus non‐fatigued samples before treatment (Figure 10c), similar to observations in single fibres from this same individual. Following KI/KCl treatment, fatigue‐induced differences in modulus were no longer evident, suggesting that differences in fatigued versus non‐fatigued passive modulus in CMT samples were dependent on intracellular mechanisms including titin. Indeed, the passive modulus following KI/KCl was dramatically reduced in all samples, highlighting the importance of intracellular mechanisms to CMT modulus in these bundles of 12–14 fibres. Though these exploratory measures with reduced sample size limit definitive conclusions in this study, previous studies of genetically modified murine muscle tissue have shown direct effects of titin on whole‐muscle passive tension (Brynnel et al., 2018), supporting the idea that alterations in titin mechanics might scale to the whole‐tissue level. Further study is required to determine how these changes manifest when scaling from in vitro cellular measures to in vivo tissue levels of organization (Ward et al., 2020). With those caveats in mind, the data in the present manuscript contribute to a growing body of literature supporting the notion that intracellular mechanisms contribute to whole‐muscle tissue mechanics in vivo, encouraging further research into the potential magnitude and dynamic regulation of these effects.

### 4.9 Limitations

The present findings support earlier data (Privett et al., 2024) suggesting a sex‐specific response to acute muscle fatigue that reduces viscoelasticity in males, but not females. These acute responses are observed regardless of the presence or absence of chronic effectors impacting viscoelasticity (physical training history; Figure 4b). Sex‐based differences in the dynamic response to acute physiological stressors are notable, but caution is warranted when attempting to extrapolate these findings to in vivo conditions. Although we examined elastic modulus in CMT samples from a subset of volunteers and demonstrated the important role of fibrillar mechanics to tissue‐level behaviours (Figure 10c), we have not replicated our primary finding in CMT. Therefore, further experiments are required to explore how altered titin‐based mechanics might impact muscle tissue that includes ECM and associated structures in vivo. Further, additional experiments are needed to clarify whether passive mechanics in permeabilized single fibres might reflect in vivo function that might impact injury prevention or performance. Indeed, although a growing body of literature supports the notion that titin plays a crucial role in stretch–shortening dynamics at the fibre level (Herzog, 2014; Herzog et al., 2016; Nishikawa, 2016, 2020; Nishikawa et al., 2013; Power et al., 2013), it is not clear at present whether alterations observed here, in passive conditions, might impact performance during dynamic loading.

## 5 CONCLUSION

In conclusion, fatiguing exercise impacts cellular passive stress and Young's modulus in a way that is mediated by training status and biological sex in young adults. Both trained and untrained young males demonstrated reduced passive stress and Young's modulus in fatigued versus non‐fatigued single fibres, whereas neither trained nor untrained young females demonstrated a change in mean stress or Young's modulus at any length. However, a look at individual differences in the mean Young's modulus of fatigued versus non‐fatigued fibres revealed that females exhibited a high degree of variability in the direction and magnitude of change in Young's modulus. We speculate that inter‐individual differences in circulating oestrogen might have contributed to this variability; however, future studies will need to measure blood oestrogen at the time of study to support or refute this idea. Like passive stress and modulus, the effect of fatiguing exercise on cellular SDI was mediated by biological sex, with only males demonstrating significantly reduced SDI in fatigued versus non‐fatigued fibres at SLs of 3.4–3.8 µm owing to changes in the magnitude of stress decay, rather than changes to the time course of stress decay. Finally, CMT assays suggest that although intracellular proteins appear to impact CMT passive modulus, their relative impact on the effect of fatiguing exercise on CMT passive mechanics is not yet clear.

## AUTHOR CONTRIBUTIONS

These experiments were conducted in the Muscle Cellular Biology Laboratory in the University of Oregon Human Physiology Department. G.E.P., A.W.R., K.W.N. and D.M.C. designed and performed experiments. G.E.P., A.W.R., K.W.N. and D.M.C. analysed and interpreted study data and revised the manuscript. G.E.P. drafted the manuscript. D.M.C. conceived and directed the study. All authors approved the final version of the manuscript and agree to be accountable for all aspects of the work in ensuring that questions related to the accuracy or integrity of any part of the work are appropriately investigated and resolved. All persons designated as authors qualify for authorship and all those who qualify for authorship are listed.

## CONFLICT OF INTEREST

None declared.

## Data Availability

The raw data that support the findings of this study are available from the corresponding author upon reasonable request.
